# Uniportal video-assisted anatomical segmentectomy: an analysis of the learning curve

**DOI:** 10.1186/s12957-023-03086-7

**Published:** 2023-07-29

**Authors:** Yu Han, Zhenrong Zhang, Hongxiang Feng, Huanshun Wen, Kunsong Su, Fei Xiao, Chaoyang Liang, Deruo Liu

**Affiliations:** 1 Department of General Thoracic Surgery, China-Japan Friendship Hospital, No. 2 Yinghua East Road, Chaoyang District, Beijing, 100029 China; 2 National Center for Respiratory Medicine, Beijing, People’s Republic of China

## Abstract

**Background:**

This study aimed to demonstrate the learning curve of anatomical segmentectomy performed by uniportal video-assisted thoracoscopic surgery (U-VATS).

**Method:**

We conducted a retrospective study of U-VATS segmentectomies performed by the same surgeon between September 2019 and August 2022. The learning curve was demonstrated using risk-adjusted cumulative sum (RA-CUSUM) analysis in terms of perioperative complications, which reflected surgical quality and technique proficiency. The surgical outcomes were also compared between different phases.

**Result:**

The complication-based learning curve of U-VATS segmentectomy could be divided into two phases based on RA-CUSUM analysis: phase I, the initial learning phase (cases 1–50) and phase II, the proficiency phase (cases 51–141). Significantly higher complication rates (24.0 vs. 8.8%, *p*=0.013), longer surgical times (119.8±31.9 vs. 106.2±23.8 min, *p*=0.005), and more blood loss (20 [IQR, 20–30] vs. 20 [IQR, 10–20] ml, *p*=0.003) were observed in phase I than in phase II.

**Conclusion:**

The learning curve of U-VATS segmentectomy consists of two phases, and at least 50 cases were required to gain technique proficiency and achieve high-quality surgical outcomes.

**Supplementary Information:**

The online version contains supplementary material available at 10.1186/s12957-023-03086-7.

## Introduction

With the results of two phase III clinical trials (CALGB140503 [1] and JCOG0802 [2]), the application of anatomical segmentectomy for the treatment of early-stage non-small cell lung cancer (NSCLC) has obtained more sufficient evidence. To minimize surgical trauma, video-assisted thoracoscopic surgery (VATS) has been widely applied in recent years [3, 4]. Recently, with the rapid development of instruments and surgical techniques, uniportal video-assisted thoracoscopic anatomical segmentectomy has been gradually developed and suggested to be a safe and feasible approach [5, 6].

As a new surgical technique, the learning curve of U-VATS segmentectomy has taken on great importance. Previous studies evaluating the learning curve were mainly based on operation time, which can reflect the speed of operation and proficiency. However, as an important indicator of surgical quality [7], the complication-based learning curve of U-VATS segmentectomy has not been fully demonstrated. In 2020, Chen et al. reported 124 cases of U-VATS segmentectomy and suggested that the learning curve consists of three phases (cases 1 to 24, cases 25 to 57, and cases 58 to 124) [8]. Similarly, Li et al. found that 64–71 cases were required to master the technique of U-VATS [9]. However, both of the studies focused on operation time rather than the complications associated with learning.

The aim of this study was to describe our experience with 141 cases of U-VATS segmentectomy and investigate the learning curve of this procedure. Cumulative sum (CUSUM) and risk-adjusted cumulative sum (RA-CUSUM) methods were used based on perioperative complications. We also evaluated the learning curves of blood loss and operation time of U-VATS segmentectomy.

### Patient and methods

#### Patient selection

From September 2019 to August 2022, 141 consecutive patients who underwent uniportal video-assisted thoracoscopic segmentectomy in the Department of Thoracic Surgery, China-Japan Friendship Hospital, were enrolled. All the cases were performed by a single team led by Dr. C.Y.L. The surgical team performed 215 cases of multiportal VATS segmentectomy before initiating U-VATS segmentectomy, most of which were two-port VATS segmentectomy.

The inclusion criteria of our study were as follows: (1) nodules suspicious for malignancy with a diameter less than or equal to 2 cm measured via CT and a consolidation/tumor ratio <0.5 in high‐resolution CT, (2) benign or metastatic tumors that were not suitable for wedge resection, (3) patients with poor pulmonary function or major comorbidities who could not tolerate lobectomy, and (4) ASA grades I–III. The exclusion criteria were as follows: (1) patients who underwent resection of more than two segments and (2) patients with a previous history of chest surgery. This study was approved by the Institutional Review Board of China-Japan Friendship Hospital (IRB-2022-KY-127: 18 July 2022). Consent of the patients for this retrospective study was waived.

#### Surgical technique

**Fig. 1:**
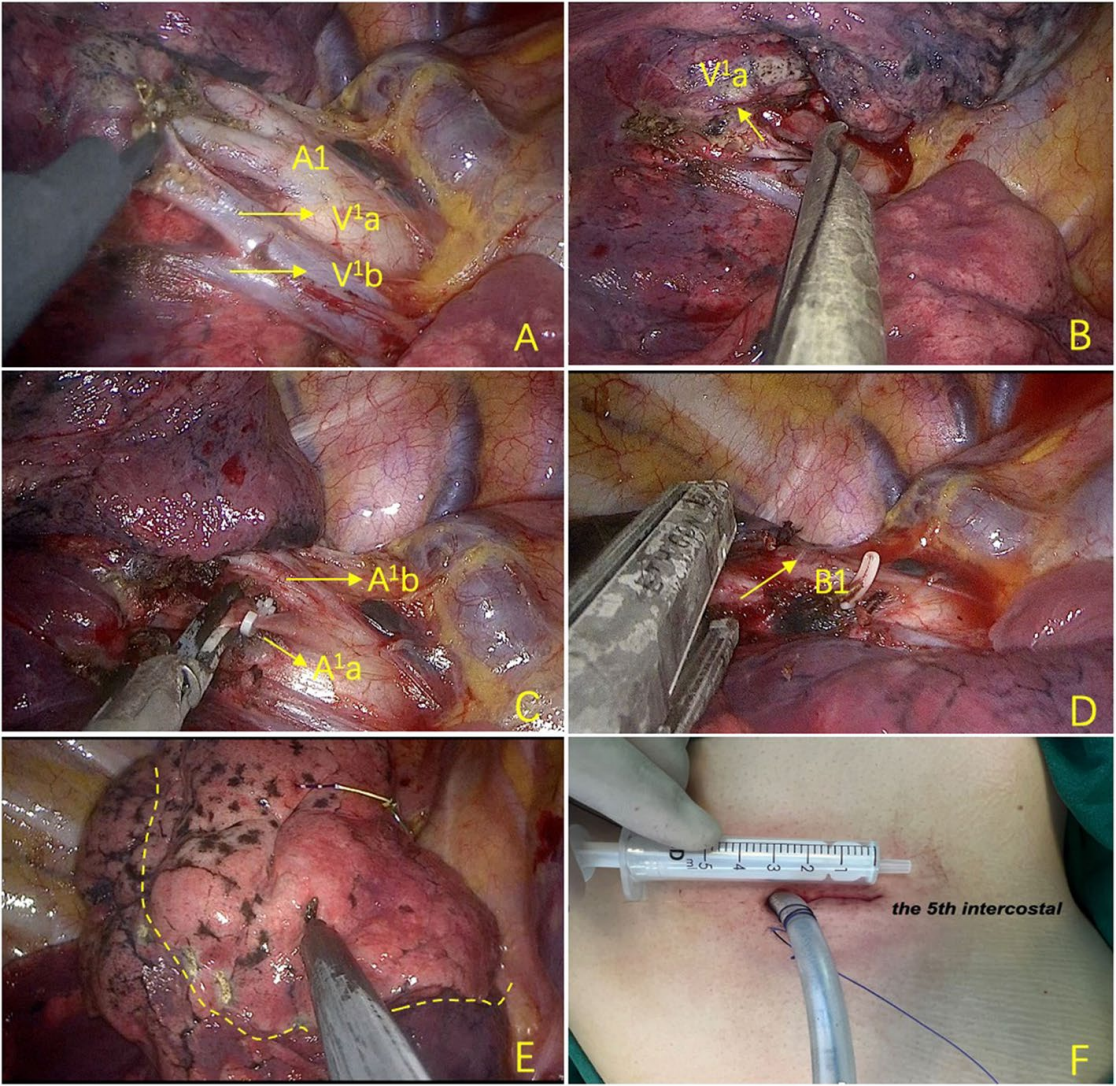
Surgical procedure of right upper lobe apical segmentectomy (S1). **A** Isolating the arteries and veins of apical segment. **B** V^1^a was ligated with silk. **C** A^1^a was clipped with Hem-o-lok. D The apical segmental bronchus (B1) was stapled. **E** Inflate-deflate technique was used to distinguish the intersegmental plane. **F** The surgical incision (S=segment; V=vein; A=artery; B=bronchus)

All patients received general anesthesia with double-lumen endotracheal intubation, and single lung ventilation was performed during surgery. All patients were placed in the lateral decubitus position, and a 3–4-cm incision was made at the fifth intercostal space on the anterior axillary line (Fig. 1F). Preoperative Hook–wire localization and three-dimensional reconstruction of CT images were selected and applied in some complex and atypical segmentectomies. The pulmonary arteries and veins of the target segment were carefully isolated and ligated during the operation (Fig. 1A–C). The lymph nodes around the bronchus were dissected, and the segment bronchus was then stapled (Fig. 1D). The intersegmental plane was identified using ventilating and deflating methods (Fig. 1E), and the parenchyma was divided by mechanical staplers. The surgical complexity of segmentectomy can be classified into simple and complex groups based on previous studies [10]. The simple segmentectomies included the right S6, left S1+2+3, left S4+5, and left S6, which had only a single linear intersegmental plane. Other segmentectomy procedures that create multiple or intricate dissection surfaces can be classified as complex procedures.

#### Statistical methods

The statistical analysis was performed with SPSS (version 23. Inc., Chicago, IL, USA). Categorical variables were compared using chi-square tests or Fisher’s exact tests. Student’s *t* test or the Wilcoxon rank sum test was used to analyze continuous variables. One-way analysis of variance (ANOVA) was employed to test continuous parametric variables with normal distributions, while the Wilcoxon rank-sum test was used to compare abnormally distributed variables. Standard deviation (SD) was used for data conforming to a normal distribution, and skewed distributions or datasets with outliers were measured with the interquartile range (IQR). A *p* value less than 0.05 was considered statistically significant.

The cumulative sum (CUSUM) method has been widely recognized and applied in the analysis of the learning curve of various operations. The CUSUM method represented the running total of the differences chronologically between the individual points and the group mean and can visually identify the consistent changes in data [11]. The CUSUM was calculated as CUSUM=$\sum_{1}^{\mathrm{n}}(\mathrm{Xi}-\upmu )$, where *xi* represents an individual operative time or blood loss, while *μ* indicates the average value of these variables.

The RA-CUSUM method [12], an extension of CUSUM, can depict complications when analyzing the learning curve. RA-CUSUM is a useful tool to reduce bias by balancing patients’ inherent risk factors for complications. The formula of RA-CUSUM was defined as $\sum_{1}^{\mathrm{n}}(\mathrm{Xi}-\uptau )+{(-1)}^{\mathrm{xi}}$ Pi, where *xi* =1 indicates the occurrence of perioperative complications, while *xi*=0 for no event; *τ* represents the observed event rate; and *Pi* stands for the expected complication occurrence based on multivariable logistic regression. We further analyzed the risk factors that may affect complications, including age, sex, BMI, FEV1%, ASA grade, tobacco use, surgical difficulty, blood loss, operative time, pleural adhesion, and lymph node dissection. First, univariable analysis was performed for all risk factors, and those with a *p* value less than 0.1 were then included in multivariable logistic regression analysis in our study.

## Results

### Basic patient characteristics

During the study period, 141 uniportal thoracoscopic segmentectomies were performed in our department by a single surgeon (Table 1), and 135 (95.7%) were malignant. R0 resections were achieved in all cases of malignant pathology. There were 47 males (33.3%) and 94 females (66.7%), with an average age of 56.1±12.9 years old. The average operative time was 110.9±27.5 min, and the median blood loss was 20 (10–20) ml. Conversion to thoracotomy occurred in two cases, and in one operation, ports were added during surgery, all of which were due to vascular injuries. Postoperative complications were observed in 20 (14.2%) patients. Eleven (7.8%) and 9 (6.4%) patients suffered grade I–II and grade III–VI complications, respectively. The average duration of drainage was 2.9±1.3 days, and the averagelength of stay was 4.0±1.9 days. The mean tumor size was 1.1±0.4 cm, and the pathology TNM stage ranged from TisN0M0 to T1cN0M0.

**Table 1:** Overall patient characteristics and perioperative outcomes (*N*=141)

Patient characteristics	
Age, years	56.1±12.9
Sex, male	47 (33.3)
BMI, kg/m^2^	23.7±2.9
Tobacco use	14 (9.9)
FEV1, in %	76.5±7.5
ASA grade	
I	99 (70.2)
II	33 (23.4)
III	9 (6.4)
Three dimensional reconstruction	71 (50.4)
Intraoperative parameters	
Operative time, min	110.9±27.5
Blood loss (IQR),ml	20 (10–20)
Vascular accident	3 (2.1)
Conversion to thoracotomy	2 (1.4)
Added port in surgery	1 (0.7)
Postoperative outcomes	
30-day morbidity	20 (14.2)
Clavien I–II	11 (7.8)
Clavien IIIa	6 (4.3)
Clavien IIIb	2 (1.4)
Clavien IV	10.7
30-Day mortality	0
Duration of drainage, day	2.9±1.3
Length of stay, day	4.0±1.9
Tumor size, cm	1.1±0.4
Pathology	
Malignant	135(95.7)
Benign	6(4.3)
R0 resection	135(100.0)
pStage	
TisN0M0	24 (17.0)
T1aN0M0	57(40.4)
T1bN0M0	54 (38.3)
T1cN0M0	6 (4.3)
LN stations	
LN1 stations (IQR)	2 (2–3)
LN2 stations (IQR)	3 (2–4)
LN numbers	
LN1 numbers (IQR)	3 (2–5)
LN2 numbers (IQR)	4 (3–6)

*BMI* body mass index, *FEV1* forced expiratory volume in 1 s, *ASA* American Society of Anesthesiologist

### Learning curve analysis

**Fig. 2:**
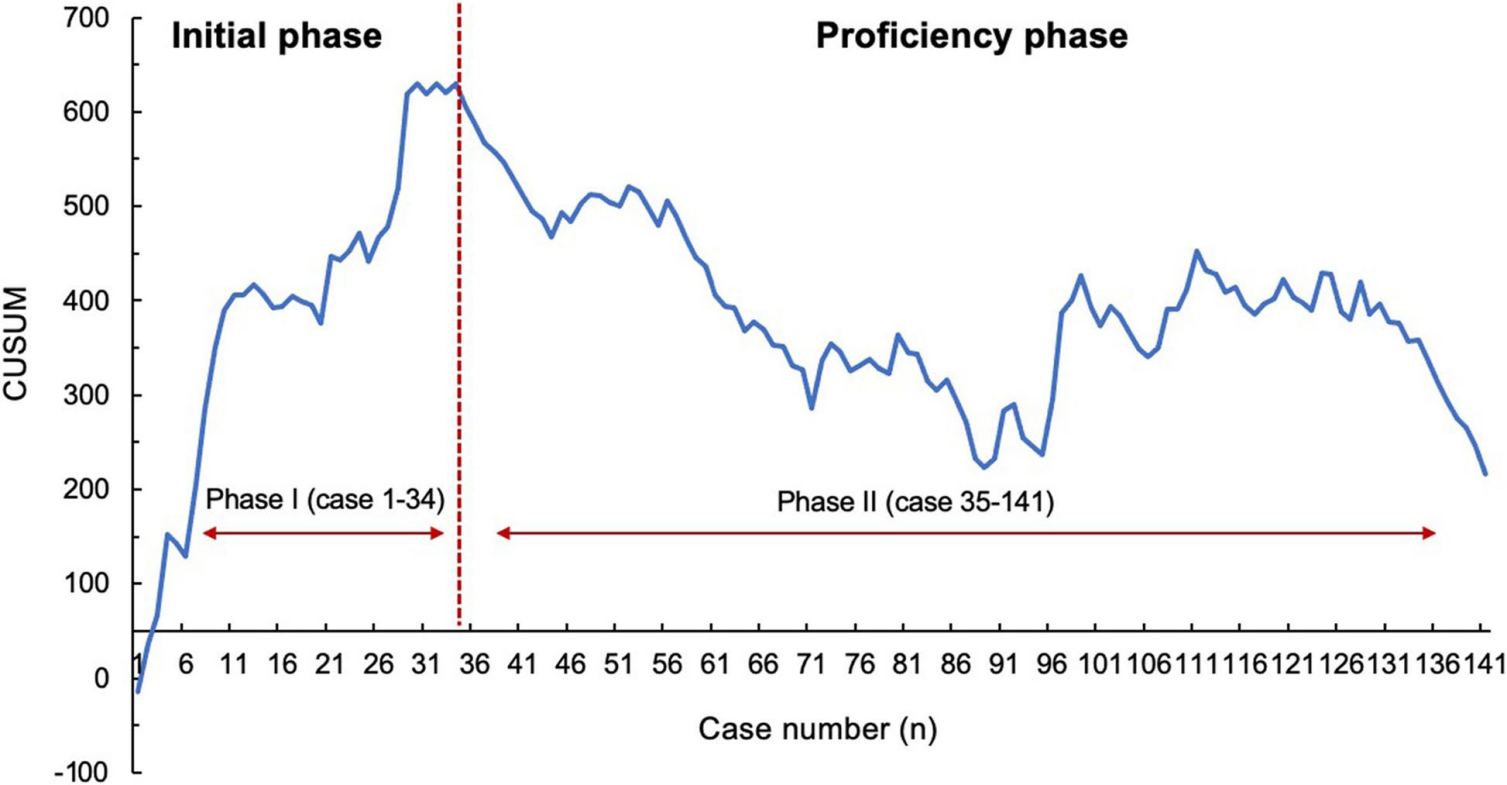
Cumulative sum analysis for operation time

**Fig. 3:**
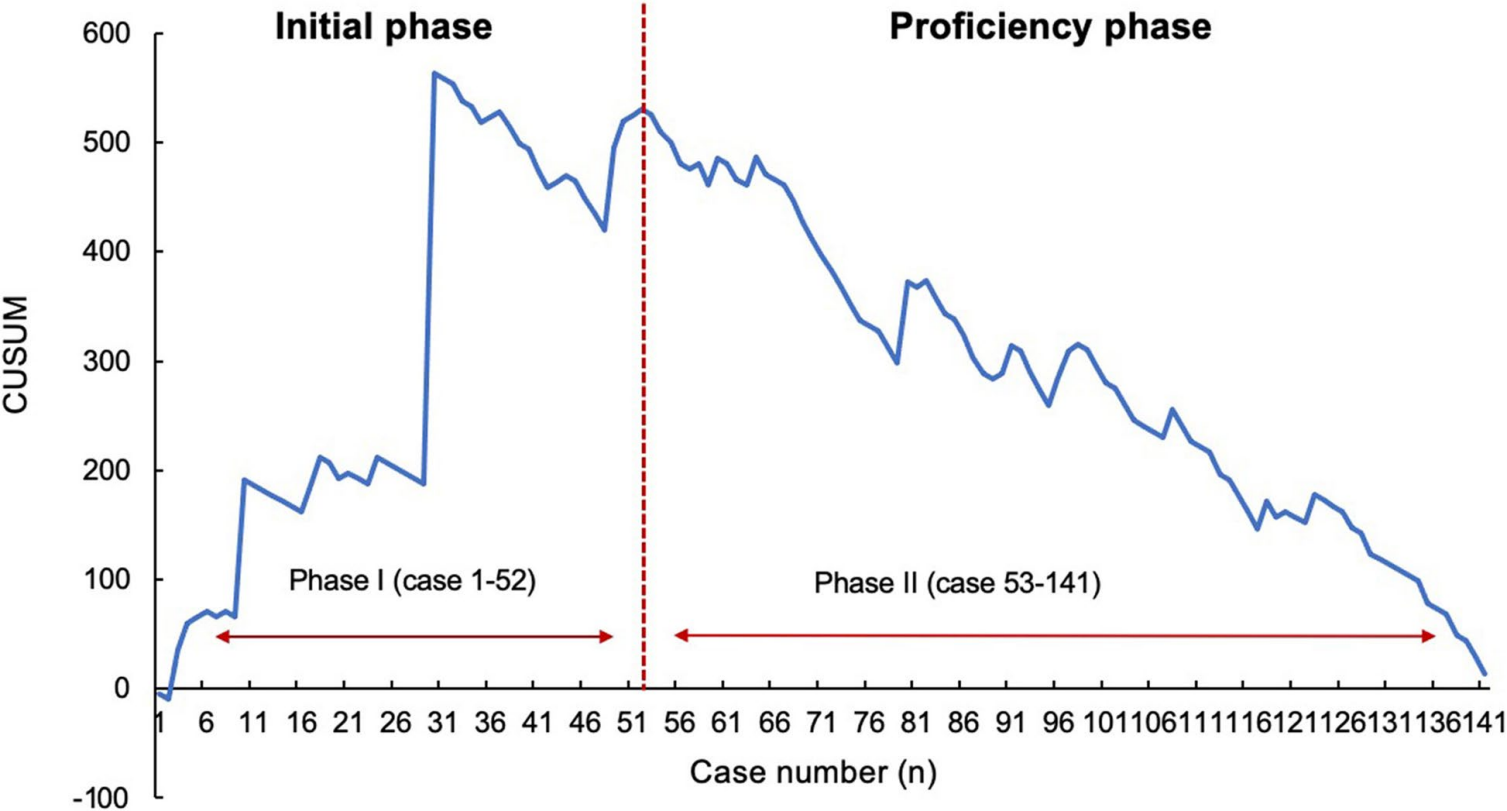
Cumulative sum analysis for blood loss

To analyze the learning curve of uniportal thoracoscopic segmentectomy, CUSUM and RA-CUSUM methods were used. The learning curve of surgical time and blood loss were initially assessed by the CUSUM method. Based on the CUSUM graph, the learning curve of operation time showed a continuous upward trend before the 34th case (cumulative summation score: 630.4), suggesting that the operative time was longer than the average level. Then, a descending trend could be observed after the 34th case, and two learning phases could be distinguished (Fig. 2). For the learning curve of blood loss, the turning point of the CUSUM graph was the 52nd case (cumulative summation score: 520.2), which demonstrated that blood loss showed a consistently descending trend after 52 cases were completed (Fig. 3).

**Fig. 4:**
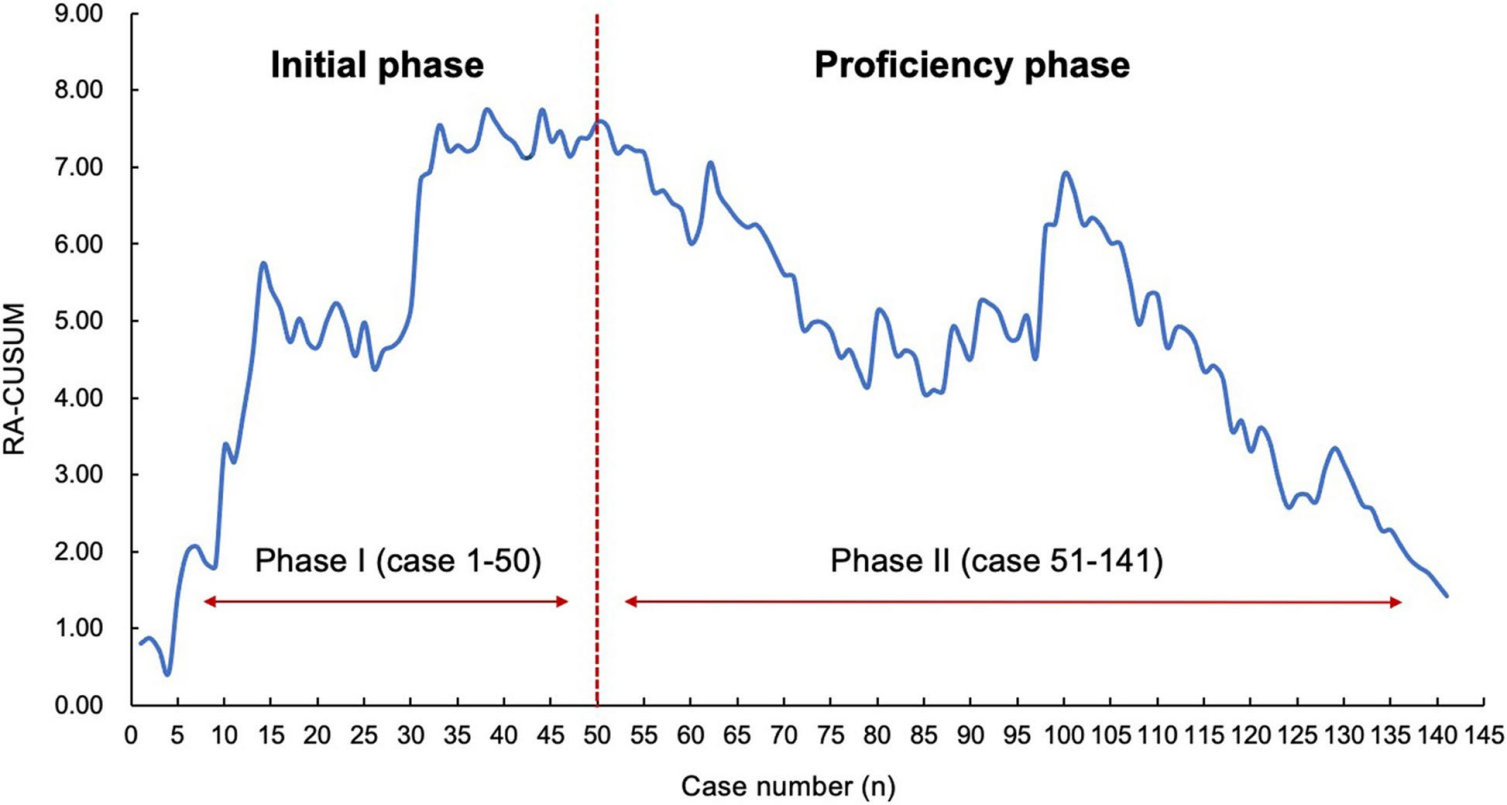
Risk adjust cumulative sum analysis for perioperative complications

To generate the complication-based learning curve of uniportal thoracoscopic segmentectomy, RA-CUSUM was performed in terms of intraoperative and postoperative complications. After univariate analysis and multivariable logistic regression, patient sex (OR, 0.129; *p*=0.010), blood loss (OR, 1.028; *p*<0.001), and operative time (OR, 1.032; *p*=0.002) were three significant independent factors for surgical complications (Supplemental Table 1). The RA-CUSUM graph showed that the change point of the learning curve was the 50th case (cumulative summation score: 7.59), which indicated that the complication rate gradually declined after 50 cases were completed and that the surgeon achieved technique proficiency (Fig. 4). Two learning phases could be identified based on RA-CUSUM analysis: phase I, the initial learning phase (cases 1–50) and phase II, the proficiency phase (cases 51–141).

### Surgical outcomes between different learning phases

The perioperative outcomes between the two learning phases were compared and are summarized in Table 2. No significant differences were observed between the different phases in terms of patient characteristics. The surgical difficulties of the cases between the two learning phases were also similar (Table 3). Compared to the initial learning phase (phase I), a significant decrease in operation time was observed (106.2±23.8 vs. 119.8±31.9, *p*=0.005) in the proficiency phase (phase II). The blood loss of phase II was also significantly lower than that of phase I (20 [10–20] vs. 20 [20–30], *p*=0.003). Moreover, the postoperative complication rate was significantly lower in the proficiency phase than in the initial learning phase (8.8 vs. 24.0%, *p*=0.013). With regard to intraoperative complications, the incidence of major vascular injury, conversion to thoracotomy, and the event of added ports were comparable between the two phases. A visual analog scale (VAS) was used to evaluate pain on postoperative day 1, and the median pain score was significantly higher in the first learning phase (4.2±1.2 vs. 3.5±1.2, *p*=0.003).

**Table 2:** Patients’ characteristics and perioperative outcomes between two learning stages

Variables	Phase I (*n*=50)	Phase II (*n*=91)	*P*
Age, years	58.0±11.9	55.1±13.5	0.209
Sex, male	15 (30.0)	32 (35.2)	0.534
BMI, kg/m^2^	23.4±2.7	23.9±3.0	0.345
FEV1/FVC, %	77.2±8.6	76.0±6.9	0.372
ASA grade (I/II/III)	34/10/6	65/23/3	0.118
History of smoking	6 (12.0)	8 (8.8)	0.565
Surgical difficulty			0.415
Easy	25 (50.0)	39 (42.9)	
Complex	25 (50.0)	52 (57.1)	
Three dimensional reconstruction	21 (42.0)	50 (54.9)	0.411
Operative time, min	119.8±31.9	106.2±23.8	0.005
Blood loss, ml	20 (20–30)	20 (10–20)	0.003
Intraoperative accidents			
Major vascular injury	2 (4.0)	1 (1.1)	0.287
Conversion to thoracotomy	1 (2.0)	1 (1.1)	1.000
Added port in surgery	1 (2.0)	0	0.355
Postoperative complications	12(24.0)	8(8.8)	0.013
Pulmonary infection	4 (8.0)	2 (2.2)	0.186
Air leak	2 (4.0)	2 (2.2)	0.615
Pleural effusion	5 (10.0)	2 (2.2)	0.097
Atrial fibrillation	0	1 (1.1)	1.000
Pulmonary embolism	0	1 (1.1)	1.000
Wound infection	1(2.0)	0	0.355
Pain score on POD1	4.2±1.2	3.5±1.2	0.003
Duration of drainage, day	3.0±1.3	2.8±1.3	0.366
Length of hospital stay, day	4.4±2.7	3.9±1.3	0.157
30-day mortality	0	0	-

*BMI* body mass index, *FEV1* forced expiratory volume in 1 s, *FVC* forced vital capacity, *ASA* American Society of Anesthesiologist, *IQR* interquartile range, *POD* postoperative day

**Table 3:** Interphase comparisons of the surgical complexity

Variable	Overall	Phase I (*n*=50)	Phase II (*n*=91)	*P*
Overall				0.763
Simple				
Left lung				
S4+5	6 (4.3)	3 (6.0)	3 (3.3)	
S6	18 (12.8)	7 (14.0)	11 (12.1)	
S1+2+3	20 (14.2)	10 (20.0)	10 (11.0)	
Right lung				
S6	19 (13.5)	6 (12.0)	13 (14.3)	
Complex				
Left lung				
S1+2	16 (11.3)	5 (10.0)	11 (12.1)	
S3	4 (2.8)	0	4 (4.4)	
S8	2 (1.4)	1 (2.0)	1 (1.1)	
S7+8+9+10	1 (0.7)	0	1 (1.1)	
S1a	1 (0.7)	0	1 (1.1)	
S1+2a	1 (0.7)	0	1 (1.1)	
S3a	1 (0.7)	0	1 (1.1)	
S8a	2 (1.4)	1 (2.0)	1 (1.1)	
S10	1 (0.7)	1 (2.0)	0	
Right lung				
S1	8 (5.7)	4 (8.0)	4 (4.4)	
S2	25 (17.7)	6 (12.0)	19 (20.9)	
S3	6 (4.3)	3 (6.0)	3 (3.3)	
S8	5 (3.5)	2 (4.0)	3 (3.3)	
S3+1b	1 (0.7)	0	1 (1.1)	
S3+2b	1 (0.7)	0	1 (1.1)	
S7+8	1 (0.7)	0	1 (1.1)	
S8a	1 (0.7)	1 (2.0)	0	
S8b	1 (0.7)	0	1 (1.1)	

## Discussion

U-VATS was first reported by Rocco in 2004 [13]. Since then, Gonzalez-Rivas successively demonstrated the technique of lobectomy, segmentectomy, and bronchial sleeve lobectomy performed by U-VATS [14–16]. To date, a series of studies have demonstrated the safety and efficacy of anatomical uniportal thoracoscopic pulmonary resection [17–19]. Xie et al. reported 1063 cases of U-VATS, including 731 cases of lobectomy and segmentectomy; no operative death occurred, and the complication rate was 5.9% [20]. Compared with open thoracotomy segmentectomy, U-VATS segmentectomy exhibited a shorter hospital stay, without any difference in terms of oncological outcomes according to the study of Surendrakumar et al. [21]. To date, many studies have been conducted to compare the surgical outcomes between U-VATS and multiportal VATS segmentectomy. The U-VATS group had potential advantages in perioperative outcomes, including shorter chest tube days, shorter hospital stays, and less postoperative pain [22]. However, because U-VATS segmentectomies are technologically more demanding than conventional multiport VATS, the learning curve should be fully demonstrated.

Previous studies that analyzed the learning curve of U-VATS segmentectomy were mainly based on operative time, and the learning curve ranged from 30 to 71 cases [8, 9, 23]. However, the surgical time alone was not sufficient for a comprehensive analysis of the learning curve. In the evaluation of surgical proficiency, other surgical outcomes, including intraoperative accidents, postoperative morbidity, mortality, and blood loss, should be considered. In addition, confounding factors should also be considered, including age, sex, BMI, ASA grade, and other factors that may affect surgical outcomes. As a consequence, the RA-CUSUM method was applied in our study to generate complication-based learning curves.

Based on our study, the perioperative complication rate was 14.2%, and no postoperative deaths occurred. Only 3 major vascular injuries occurred in the case series, conversion to thoracotomy occurred in 2 cases, and adding a port occurred in 1 case. Based on RA-CUSUM analysis, the learning curve could be divided into two phases regarding complications. We demonstrated that 50 cases were required to obtain improved surgical outcomes. Furthermore, the surgical time was significantly reduced after 34 cases were completed, while the turning point of blood loss was the 52nd case.

In general, surgeons may avoid complex surgeries when initially implementing the new surgical technique to achieve better surgical outcomes [24]. However, the surgical complexity was similar between the different learning phases in our study, which reduced the bias to a certain extent. The postoperative complication rate was significantly higher in the initial learning phase than in the experienced phase (24.0% vs. 8.8%, *p*=0.013), which may be attributable to the longer operative time and more unnecessary tissue damage during the initial learning phase. Because all the instruments were placed through a single incision during surgery, surgical instruments are likely to collide and interfere with each other, which may cause compression of the incision and enhance postoperative pain. To solve this problem, we used elongated surgical instruments and double-joint surgical instruments after we performed approximately 10 cases. The interference between instruments was significantly reduced, which was helpful to shorten the operation time.

The limitation of this study should also be considered. First, our research only analyzed the learning curve of a single surgeon, and multicenter studies are necessary to fully demonstrate the learning curve of U-VATS segmentectomy in the future. Second, our surgical team had extensive surgical experience with multiportal VATS segmentectomy and traditional open segmentectomy. Therefore, our study may have more guiding value for surgeons with experience in VATS but not novice surgeons without experience in VATS. Third, three-dimensional reconstruction was applied in some of the surgeries in our case series, which may have influenced the learning curve of operative time. In addition, the speed of operation and surgical quality could be affected by the experience of assistants, which may also affect the learning curve. Fourth, the number of segmentectomies in the lower lobe (*n*=51) was smaller than that in the upper lobe (*n*=90) in our study. One possible reason was that perhaps more patients with lesions in the lower lobe were offered wedge resection or lobectomy, which led to this bias. The learning curve may be more precise if more segmentectomies in the lower lobe were added. Finally, quality of life (QoL) assessment after surgery is necessary to generate the learning curve. Although postoperative pain was recorded in our study, we did not evaluate QoL with a standard questionnaire such as the EuroQol five-dimensional questionnaire (EQ-5D). Based on our experience, patients would obtain higher quality of life scores after the learning curve has been overcome because the complication rate would decrease dramatically. The adverse reactions of anesthesia will also decrease with a shortening of the operation time. Further study is needed regarding the changes in QoL during the learning curve.

## Conclusions

In conclusion, our research demonstrated that the U-VATS segmentectomy technique was feasible, and at least 50 cases were required to overcome the complication-based learning curve.

### Supplementary Information

**Additional file 1.**

**Additional file 2:** **Supplemental Table 1.** Univariable and multivariable analyses of the risk factors of perioperative complications.

## Data Availability

Data can be provided upon reasonable request from the corresponding author.
