# Correction: Uniportal video-assisted anatomical segmentectomy: an analysis of the learning curve

**DOI:** 10.1186/s12957-023-03135-1

**Published:** 2023-08-08

**Authors:** Yu Han, Zhenrong Zhang, Hongxiang Feng, Huanshun Wen, Kunsong Su, Fei Xiao, Chaoyang Liang, Deruo Liu

**Affiliations:** 1 Department of General Thoracic Surgery, China-Japan Friendship Hospital, No. 2 Yinghua East Road, Chaoyang District, Beijing, 100029 China; 2 National Center for Respiratory Medicine, Beijing, People’s Republic of China

**Correction: World J Surg Onc 21, 232 (2023)**

**https://doi.org/10.1186/s12957-023-03086-7**

Following publication of the original article [1], the author wants to add another funding to the article:

*Elite Medical Professionals Project of China-Japan Friendship Hospital (NO.ZRJY2021-GG07).*

The original article has been updated.
