# Supplementary material for: Uniportal video-assisted anatomical segmentectomy: an analysis of the learning curve
Source: World J Surg Oncol. 2023 Jul 29;21:232. doi: 10.1186/s12957-023-03086-7 (PMC10386600; doi:10.1186/s12957-023-03086-7)
Supplement: Supplementary file 2 — Additional file 2: Supplemental Table 1. Univariable and multivariable analyses of the risk factors of perioperative complications. [file 12957_2023_3086_MOESM2_ESM.docx]

**Supplemental Table 1. Univariable and multivariable analyses of the risk factors of perioperative complications**

| Variables | Without Complications (n=121) | With  Complications  (n=20) | Univariate analysis | multivariable analysis | | |
| --- | --- | --- | --- | --- | --- | --- |
|  |  |  | *P* Value | B | P value | OR |
| Age, years | 54.9±12.9 | 62.3±11.6 | 0.012 |  |  |  |
| Sex, male | 34(28.8) | 13(56.5) | 0.010 | -2.049 | 0.001 | 0.129 |
| BMI, kg/m^2^ | 23.6±2.8 | 24.3±3.4 | 0.010 |  |  |  |
| FEV1/FVC, % | 77.2±7.0 | 72.1±9.1 | 0.284 |  |  |  |
| ASA grade (I/II/III) | 85/28/5 | 14/5/4 | 0.061 |  |  |  |
| History of smoking | 9(7.6) | 5(21.7) | 0.064 |  |  |  |
| Surgical difficulty |  |  | 0.822 |  |  |  |
| Simple | 53(44.9) | 11(47.8) |  |  |  |  |
| Complex | 65(55.1) | 12(52.2) |  |  |  |  |
| Blood loss, ml | 20(20-50) | 20(10-20) | ＜0.001 | 0.028 | 0.035 | 1.028 |
| Operative time, min | 106.5±23.2 | 134.0±35.8 | 0.002 | 0.032 | 0.001 | 1.032 |
| Pleural adhesion | 6(5.1) | 3(13.0) | 0.336 |  |  |  |
| Tumor size, cm | 1.1±0.4 | 1.2±0.5 | 0.276 |  |  |  |
| LN1 stations | 2(2-3) | 2(1-3) | 0.655 |  |  |  |
| LN2 stations | 3(2-4) | 3(2-4) | 0.203 |  |  |  |
| LN1 numbers | 3(2-5) | 4(2-4) | 0.894 |  |  |  |
| LN2 numbers | 4(3-6) | 4(3-6) | 0.562 |  |  |  |
| Constant |  |  |  | -5.034 | ＜0.001 | 0.007 |

BMI: Body mass index, FEV1: Forced expiratory volume in 1 second, FVC：forced vital capacity, ASA: American Society of Anesthesiologist, LN: Lymph node.
